# Methodology for Evaluating the Insect Growth Regulator (IGR) Methoprene on Packaging Films

**DOI:** 10.3390/insects7030033

**Published:** 2016-07-07

**Authors:** Frank H. Arthur

**Affiliations:** Center for Grain and Animal Health Research, Agricultural Research Service, USDA, 1515 College Avenue, Manhattan, KS 66502, USA; frank.arthur@ars.usda.gov; Tel.: +1-785-776-2783

**Keywords:** methoprene, stored product insects, residual, control

## Abstract

The insect growth regulator methoprene can be mixed into the matrix used to comprise bags and other packaging materials. Different methodologies were utilized to evaluate the efficacy of different types of methoprene-treated packaging towards *Tribolium castaneum* (Herbst), the red flour beetle, and *T. confusum* Jacquelin duVal, the confused flour beetle, two common insect species that infest stored products. Tests were conducted by creating arenas in which larvae were exposed on the packaging surface along with a flour food source, and assessments were made on adults emerging from the exposed progeny. Tests were also done by exposing adults, again with a flour food source, removing the adults after one week, and assessing adult emergence of progeny from those parental adults. In tests with larvae exposed on methoprene-treated birdseed bags, the outside surface had more activity compared to the inside surface, especially on *T. confusum*. In other studies with different types of packaging materials, there was generally 100% inhibition of adult emergence of exposed larvae or of progeny adults when parental adults were exposed on the methoprene-treated packaging. The best technique for evaluation was to expose late-stage larvae as the test life stage. Results show the potential of using methoprene-treated packaging for bagged storage of processed grains and grain products.

## 1. Introduction

The insect growth regulator (IGR) methoprene is labelled in the United States of America (USA) for use as a grain protectant, a residual surface treatment, and as an aerosol for structural treatment to mills and processing plants. The application rates of 1.25 and 2.5 ppm of the current commercial product Diacon^®^ IGR (Central Life Sciences, Schaumberg, IL, USA) will give control of externally-feeding stored product insect pests on different stored grains for at least two years [1]. When immature stages of *Tribolium* species are exposed to methoprene, they can be arrested in that stage, exhibit incomplete development between life stages, or emerge as adults with various morphological deformities such as twisted or incomplete wings, unsclerotized body segments, deformed body parts including legs, and incomplete abdominal segmentation [2]. Methoprene is persistent on different flooring surfaces, though residual efficacy can vary with the type of surface [3]. Methoprene can also be applied as an aerosol, but in the USA it is usually combined with pyrethrins or a pyrethroid [4]. When methoprene is applied in combination with pyrethrin as a surface treatment or as an aerosol, the residual deposits will give control of *Tribolium castaneum* (Herbst), and *Tribolium confusum* Jacqueline duVal, for weeks or even several months [4,5].

Residual surface treatments and aerosols, including methoprene, are valid components of insect pest management programs for facilities that manufacture, store, or distribute processed grain products. Aerosols are being increasingly considered as alternatives to fumigants, including methyl bromide, because they are generally safer to use than fumigants and do not require the long shut-down times associated with fumigants [6]. A new utilization of methoprene in the USA is by incorporation into the adhesive packaging matrix of bags used to store processed and manufactured food products. The commercial bagging material is registered by and marketed through Provision Gard Inc. (Greensboro, NC, USA; pvgard.com).

Both *T. castaneum* and *T. confusum* can feed on and infest a wide variety of food products. Currently, there are few published scientific studies reporting on the efficacy of methoprene as a packaging treatment. Therefore, the objectives of this study were to evaluate efficacy of methoprene mixed into the laminate layers of different packaging films and laminates, using either one or both of the above species. Several different studies were conducted, each of which will be described separately.

## 2. Materials and Methods

### 2.1. Experiment 1

Animal feed bags in which 0.1% active ingredient (AI) methoprene had been mixed into the packaging laminate matrix were received by the USDA-ARS-Center for Grain and Animal Research (CGAHR) to be used in the initial test. The bags had been manufactured about one month prior to their arrival at the CGAHR. Circular sections of the bagging material were cut to fit the bottom portion of Plastic Petri dishes (100 by 15 mm lid measurement, 62 cm^2^ for the bottom portion). Arenas were created so that either the outside or the inside of the bag was the exposure surface. The discs were placed into the individual Petri dish bottoms, then caulked around the edges to seal those edges and thus deter larvae from crawling underneath the surface. The experimental design was that residual efficacy of the bags was to be evaluated at 2-month intervals, and the initial test was termed the 0-month test. For this test, there were 12 replicates each of the outside and inside surface discs cut from the treated bags. These were subdivided into two groups, half were used for bioassays with *T. castaneum* and the other half for bioassays with *T. confusum*. Approximately 300 mg of flour was placed in each arena to provide a food source for the larvae, and then ten 4-week-old *T. castaneum* larvae were placed in each of the six arenas containing either the outside or inside of the bag as the exposure surface. This process was repeated for *T. confusum*. All insects were obtained from pesticide-susceptible colonies that had been reared at the CGAHR for more than 30 years. Bags had also been received that had not been treated with methoprene, these were used for untreated controls. An equal number of control arenas were created, again using both the outside and inside of the bag surfaces, as described for the treated bags. The bags were then stored in a closed cardboard box under ambient conditions in an outer building at the center, which was unlit by artificial light except when personnel entered the room. This room was not climate-controlled.

After the larvae were placed in the arenas, those arenas were then placed in an incubator set at 27 °C-60% RH (relative humidity). The criteria for assessment was the emergence of adults that were normal in morphological appearance without any visible defects, as described earlier. The test was concluded approximately two weeks after adult emergence had been completed in untreated controls. Every two months, new exposure arenas were created as described above, using the bagging material stored in the outside shed. Data were analyzed using the REG Procedure of the Statistical Analysis System (Version 9.1, SAS Institute, Cary, NC, USA) to determine if adult emergence followed an ordered pattern or sequence during the 12-month residual test. Data were then analyzed by using the *t*-test Procedure (PROC *t*-test) to determine differences between adult emergence on the outside *versus* the inside of the bag, and for emergence of *T. castaneum* compared to *T. confusum* on either bag surface.

### 2.2. Experiment 2

In this test, experimental arenas were created in bottom portions of square 100 by 15 mm (top dimension) plastic Petri dishes (approximately 62 cm^2^ total area), using two different materials with 0.1% AI methoprene that had been mixed into the laminate matrix during the manufacturing process. The bags had been manufactured about 3 months prior to being received at the CGAHR Center. The first material was one in which the entire end tab from the bag had been treated with methoprene. A section of the bag was cut into a 9 cm by 9 cm square, and placed into the bottom of a Petri dish, inside surface down so that the outside surface was exposed, then caulked around the edges, again to deter larvae from crawling underneath the surface. The actual treated portion of the square cut from the bag was about 27 cm^2^, or 33% of the total area. The second material was one in which only the end tab thread had been treated with methoprene. A section measuring approximately 9 cm by 6 cm was cut from the bag, placed in the bottom portion of the Petri dish, and then caulked around the edges. A total of 40 treated arenas were created, along with an equal number of untreated controls that were cut from bags manufactured without the treated thread. 

For both materials, a test was initiated approximately 1 week after the bagging materials were received, by exposing 10 adult *T. castaneum* in each of five replicate arenas created for each of the two bagging materials (end tab treated and thread treated), along with five replicates of untreated control arenas. Each arena contained about 2 g of whole-wheat flour to provide the food source for the F_1_ progeny. The process was repeated for *T. confusum*. These arenas were then placed in an incubator set at 27 °C-60% RH, held for a week, and then the adults were removed from the arenas. After an additional 6 weeks, the arenas were removed from the incubator and the number of progeny adults emerging from eggs laid by the exposed parental adults was tabulated. The entire exposure process was repeated again about 3 months after the first trial, using the remaining arenas created before the first trial. Adults were exposed in the arenas for 1 week, removed, and the arenas were held to determine adult progeny as described above. Only the outside surface was evaluated in this study because of the design of the packaging material. Data were analyzed by using the *t*-test Procedure of SAS to determine differences in adult progeny development between the two species.

### 2.3. Experiment 3

A series of tests was then conducted evaluating efficacy of methoprene mixed into the laminate layers of different packaging materials, using only 4-week-old larvae of *T. castaneum* as the test life stage. Again, because of the design of those materials, only the outside surface was evaluated. For all these tests, the experimental unit was a circular piece of the bagging material cut to fit the 62 cm^2^ area of the round Petri dish bottom, as described in Experiment 1. Each test will be described in separate paragraphs.

This first test involved four different packaging types: 0.2% AI methoprene ink strip card stock treated with methoprene, and 0.25%, 0.1%, and 0.2% methoprene applied into the laminate matrix of the card stock paper without any ink. Sections measuring approximately 7.6 by 5.1 cm were cut from each packaging type and caulked to the bottom portion of the 62 cm^2^ plastic Petri dish. Five replicate arenas were created for each treatment, along with five replicate arenas each of the ink strip paper and card stock paper that did not contain methoprene (untreated controls, 10 total). Ten 4-week-old *T. castaneum* larvae, along with 300 mg of flour, were placed in each treated and untreated arena. The arenas were held at 27 °C-60% RH until adult emergence was completed on the untreated controls.

The second test was three different thin candy bar wrappers, termed sets 1, 2, and 3. These wrappings essentially consist of a thin outer layer, middle layer termed the adhesive layer, and an inner layer called the “cold sealed” layer. This inner layer is closest to the contents of the package, and the adhesive layer seals the outer and inner layers together. Set 1 had 0.2% AI methoprene mixed into the primer adhesive layer of the wrapping, set two was the same treatment to the adhesive layer plus 0.2% AI methoprene applied into the cold sealed layer, in set 3 only the cold sealed inner layer was treated with 0.2% AI methoprene. Larval and adult exposure arenas were created using material from each set, five replicates for larvae arenas and five replicates for adult exposure arenas. No untreated material could be provided, hence filter paper was used for the untreated controls (five replicates) for larvae and adult exposures. Arenas were created by cutting rectangular pieces of material, then caulking the edges to deter larvae from crawling underneath during the exposure period.

The final test was to commercial packaging products used in the construction of birdseed bags. The first is called PE to PE, the outer layer is polyethylene (1.25 mil thickness) that has been treated with 0.1% methoprene, printed and adhered to the inner layer of the same type of material (PE). The second is called PE to EVOH, the outer layer is polyethylene (1.25 mil thickness) that had been treated with 0.1% methoprene, printed and adhered to the inner layer of EVOH which is a 5-layer blown film. One question regarding the construction of this bag is whether or not the methoprene would diffuse through the EVOH material to the inside of the bag. In this test, both the larval exposure methodology and the adult exposure methodology described in Experiment 2 were employed, using *T. castaneum* only as the test species. For the larval exposures, the treatments were the outside and inside layers of each of the two materials, with five replicates. Arenas were created as previously described, and 10 late-instar larvae were added to each arena along with about 300 mg of flour as a food source for the larvae. No untreated material was provided for this test because it was not commercially available. Thus, untreated controls were exposed in arenas containing filter paper (5 replicates). The larval arenas were held at 27 °C-60% RH.

For the adult exposures, the same number of arenas were created for the two treatments and the outside and inside surfaces. Ten 1–2-week old adults were exposed with 2 g of flour in each arena, again to provide a food source for developing progeny. The arenas were held for one week at 27 °C-60% RH, the adults were removed, and the arenas returned to the incubator for 8 weeks to assess development of progeny to the adult stage. Again, no untreated material could be provided, hence filter paper was again used for untreated controls, prepared and processed as described for the treated arenas.

## 3. Results

### 3.1. Experiment 1

There was variation in the percentage of morphologically-normal adults emerging from the exposed larvae of both *T. castaneum* and *T. confusum* during the 12-month residual study (Table 1). However, there was no ordered pattern, and regressions with month as the independent variable and adult emergence of either species exposed on either the inside or outside of the packaging material were not significant (*p* ≥ 0.05). Thus, data were compared by using the two-sample t-tests to compare results between species and between the outside of the packaging surfaces. On both surfaces, it was clear that *T. castaneum* was more susceptible to the methoprene bagging material compared to *T. confusum*, with significantly lower adult emergence on 9 of 14 possible comparisons. Similarly, it was also evident that the outside portion of the bagged packaging had a higher level of activity compared to the inside surface, with lower adult emergence on 5 of 7 comparisons for *T. castaneum* and 7 of 7 comparisons for *T. confusum*.

### 3.2. Experiment 2

The number of progeny adults of *T. castaneum* and *T. confusum* in the untreated controls (averaged over the two treatments, end-tab treated and thread treated, and the 0 and 3-month bioassays) was 30.1 ± 3.5 for *T. castaneum* and 20.4 ± 1.9 for *T. confusum*, respectively. No progeny adults emerged at either 0 or 3 months in the arenas containing the treated end tabs or the treated thread, indicating 100% suppression of progeny.

### 3.3. Experiment 3 Series

No adult *T. castaneum* emerged from the larvae exposed on the ink stock paper or the plain card paper. Adult emergence in the ten untreated controls averaged 73.3% ± 5.5%. Adult emergence from the larvae exposed on the three candy bar wrappers termed set 1, set 2, and set 3 was 36.0% ± 18.1%, 0, and 0, respectively. Adult emergence in the untreated controls with filter paper was 82.5% ± 10.0%. No progeny adults were recorded on any of the candy bar wrappers, while progeny adult emergence on the filter paper averaged 59.8 ± 4.1. Adult emergence from *T. castaneum* larvae exposed on the outside and the inside of the PE-PE bags was 2.0 ± 2.0, and 0, respectively. The percentage of adults emerging from larvae exposed on the outside and inside of the PE-EVOH bags was 4.0% ± 2.4%, and 20.0% ± 11.0%, respectively. Adult emergence in untreated controls was 90.0% ± 4.1%. For the second part where adults were exposed in the arenas for one week and then removed, after 2 months there was one adult on one of the five replicate PE-EVOH outside arenas and one adult on one of the five replicate PE-PE inside arenas. These could have been adults that had been missed when the original parental group was removed from the arena. Adult progeny of *T. castaneum* in untreated controls with the filter paper averaged 17.0 ± 1.7.

## 4. Discussion

Susceptibility of *T. castaneum* and *T. confusum* varies depending on the specific insecticide, life stage, and exposure conditions [7]. In previous studies whereby methoprene has been applied as a liquid spray as a residual surface treatment, or as an aerosol in combination with pyrethrin, larval *T. castaneum* were more susceptible than *T. confusum* [8]. Similar results were obtained in the 12-month residual test described here, especially with the inside surface of the packaging. However, these studies cited above all used the same laboratory strains of both species, one that had been reared in the laboratory for more than 30 years. Field strains of stored-product insects vary in susceptibility to insecticides, and may differ from laboratory strains [9,10]. Thus, the results reported here may not be precisely transferred to field strains of *T. castaneum* and *T. confusum*, but they do provide general indications of the efficacy of methoprene-treated packaging.

The results of the test in which adults were exposed on untreated surfaces for one week, and assessments were made on progeny production, were variable and gave inconsistent results. There was low progeny production for untreated controls in the treated end tabs, treated thread, which utilized untreated material of the same type used in the test, and untreated controls on filter paper used in the PE-PE and PE-EVOH tests, but consistent progeny production on the filter paper used in the candy wrapper test. In a previous study evaluating methoprene and the chitenase-inhibitor novoluron, the same methodology was employed whereby adult *T. castaneum* were exposed for a week on arenas comprised of concrete, with 2 g of flour, and the adults removed after 1 week, with relatively low progeny production on that surface as well [11]. Collecting known numbers of eggs to assess effects of methoprene throughout the entire life stage may be an option, but eggs are fragile and easily damaged, in addition collecting large numbers of eggs is very time-consuming. A possible alternative would be to expose larvae of different age ranges, which may provide a more realistic measure of susceptibility than just exposing one larval life stage.

Methoprene inhibited adult emergence of larval *T. castaneum* on a wide variety of different packing films and construction material evaluated in the current study. Only one test involved evaluations on the inside surface of the packaging material, with noticeable loss of efficacy on the inside surface, especially with *T. confusum*. Currently, there is a need to examine in more detail the susceptibility of other stored product insect species to methoprene-treated packaging. Recent studies indicate that larval *Trogoderma variabile* (Ballion), the warehouse beetle, are more tolerant to methoprene compared to *T. castaneum*, especially on the inside surface of the bag material [12]. *Plodia interpunctella* (Hübner), the Indianmeal moth, is also a damaging insect pest of stored products commonly found in pet foods [13], and neonates can penetrate sound packaging [14]. Larvae of *P. interpunctella* are susceptible to methoprene applied as an aerosol [15,16], and research on the susceptibility of larvae to different packaging materials containing methoprene would be of great value to the food storage industries.

## 5. Conclusions

Methodologies have been developed for manufacturing packaging films treated with the insect growth regulator methoprene. Bioassays can be conducted to determine susceptibility of immature life stages of stored product insects exposed on these different packaging materials. A variety of packaging materials were tested in different studies, using *T. castaneum* and *T. confusum* as the test insects. Exposing late-stage larvae was the preferred method for assessing efficacy compared to exposing adults and evaluating resulting progeny production.

## Figures and Tables

**Table 1:** Percentage (mean ± SE) of adult emergence of 4-week old larvae of *T. castaneum* and *T. confusum* exposed on the outside and inside surfaces of methoprene-treated bags (0.1% active ingredient). Bioassays were done every two months for 12 months. Emergence of morphologically normal adults in untreated controls of both species was above 95%, data eliminated from analysis ^1^.

Species	Month	Outside (%)	Inside (%)
*T. castaneum*	0	0.0 ± 0.0 ^a,A^	5.0 ± 2.2 ^a,B^
	2	0.0 ± 0.0 ^a,A^	1.7 ± 1.7 ^a,B^
	4	3.3 ± 2.1 ^b,B^	18.3 ± 3.1 ^a,B^
	6	3.3 ± 3.3 ^b,A^	66.7 ± 21.8 ^a,A^
	8	0.0 ± 0.0 ^b,B^	25.0 ± 12.3 ^a,B^
	10	0.0 ± 0.0 ^b,A^	13.3 ± 4.9 ^a,B^
	12	1.7 ± 1.7 ^b,B^	70.0 ± 5.8 ^a,B^
*T. confusum*	0	6.7 ± 6.7 ^b,A^	41.7 ± 14.7 ^a,A^
	2	5.0 ± 2.3 ^b,A^	26.7 ± 14.1 ^a,A^
	4	20.0 ± 6.3 ^b,A^	65.0 ± 4.6 ^a,A^
	6	0.0 ± 0.0 ^b,A^	81.7 ± 4.0 ^a,A^
	8	11.7 ± 4.1 ^b,A^	95.0 ± 3.4 ^a,A^
	10	5.0 ± 3.4 ^b,A^	93.3 ± 4.2 ^a,A^
	12	38.3 ± 7.5 ^b,A^	83.3 ± 2.1 ^a,A^

^1^ Means between columns for adult emergence on outside *versus* inside surface significant when denoted by different lower case letters. Means between each data point for adult emergence of *T. castaneum* compared to *T. confusum* at each bi-monthly bioassay on each bag surface are denoted by different capital letters (*p* < 0.05, PROC *t*-test, SAS Institute).
